# Developmental Morphology and Anatomy Shed Light on Both Parallel and Convergent Evolution of the Umbellate Inflorescence in Monocots, Underlain by a New Variant of Metatopy

**DOI:** 10.3389/fpls.2022.873505

**Published:** 2022-04-29

**Authors:** Jesús Martínez-Gómez, Tara A. M. Atluri, Irving Jason Rose, Aaliyah J. Holliday, Christopher F. Strock, Jonathan P. Lynch, William B. Miller, Dennis Wm. Stevenson, Chelsea D. Specht

**Affiliations:** ^1^Section of Plant Biology and the L.H. Bailey Hortorium, School of Integrative Plant Science, Cornell University, Ithaca, NY, United States; ^2^Department of Plant Science, Pennsylvania State University, University Park, PA, United States; ^3^Section of Horticulture, School of Integrative Plant Science, Cornell University, Ithaca, NY, United States; ^4^New York Botanical Garden, Bronx, NY, United States

**Keywords:** inflorescence, meristems, metatopy, monocots, Evo-Devo, convergent evolution, developmental biology, developmental system drift

## Abstract

Inflorescence structure is very diverse and homoplasious, yet the developmental basis of their homoplasy is poorly understood. To gain an understanding of the degree of homology that these diverse structures share, we characterize the developmental morphology and anatomy of various umbellate inflorescences across the monocots and analyzed them in an evolutionary context. To characterize branching order, we characterized the developmental morphology of multiple inflorescences with epi-illumination, and vascular anatomy with Laser Ablation Tomography, a novel high-throughput method to reconstruct three-dimensional vasculature. We used these approaches to analyze the umbellate inflorescences in five instances of presumed homoplasy: in three members of the Amaryllidaceae; in three members of the Asparagaceae, including a putatively derived raceme in *Dichelostemma congestum*; in *Butomus umbellatus* (Alismataceae), in *Tacca chantrieri* (Dioscoreaceae), and in umbellate structure in *Fritillaria imperialis* (Liliaceae). We compare these with racemes found in three members of the subfamily Scilliioideae (Asparagaceae). We find there are three convergent developmental programs that generate umbellate inflorescences in the monocots, bostryx-derived, cincinnus-derived and raceme-derived. Additionally, among the bostryx-derived umbellate inflorescence, there are three instances of parallel evolution found in the Amaryllidaceae, in two members of Brodiaeoideae (Asparagaceae), and *Butomus umbellatus*, all of which share the same generative developmental program. We discuss the morphological modifications necessary to generate such complex and condensed structures and use these insights to describe a new variant of metatopy, termed horizontal concaulesence. We contextualize our findings within the broader literature of monocot inflorescence development, with a focus on synthesizing descriptive developmental morphological studies.

## Introduction

The mechanism by which developmental variation underlies novel and adaptive morphologies is a key research program in evolutionary and developmental (Evo-Devo) biology (Moczek et al., 2015). Under the structuralist framework (see Charlesworth et al., 1982) for the neo-darwinian perspective), the developmental morphological hierarchy represents a source of biological variation which serves as a substrate for evolutionary forces (Gould and Eldredge, 1977; Smith et al., 1985). Detailed comparative developmental morphological studies are necessary to characterize evolutionary mechanisms and can elucidate fundamental principles in organismal biology (Kaplan, 2001).

To fully understand the role of developmental variation in the evolution of form and function, it must be investigated within a comparative phylogenetic framework. Convergent evolution and parallelism are two key outcomes of particular interest to evolutionary biologists and can only be interpreted in a phylogenetic context. Parallel evolution, *sensu* M. H. Wake, is a macroevolutionary manifestation of evolutionary forces independently acting upon a homologous generative developmental program (Wake, 2015). In contrast, convergent evolution where evolutionary forces independently act on non-homologous generative developmental programs. Discriminating between parallel evolution and convergent evolution can shed light on how biological variation, at the developmental level, produces novel, adaptive or otherwise evolutionary significant morphologies (Wake et al., 2011; Brigandt and Love, 2012; Wake, 2015): The developmental morphological characterization of a homoplasious character, *sensu* Nixon and Carpenter, will shed light onto the mechanistic origins underlying the origins of phenotypic novelty and organismal diversity (Kaplan, 2001; Nixon and Carpenter, 2012; Moczek et al., 2015; Wake, 2015).

Inflorescence diversity within the monocots provides an excellent system to investigate the role of developmental morphology in evolutionary outcomes with functional implications. Inflorescences are modified shoots where flowers originate on, in angiosperms. They are distinct from the vegetative shoot, typically demarcated by one or more morphological shifts in phyllotaxy, internode length and leaf morphology (Weberling, 1992). Inflorescences are important to plant reproduction and play a key role in population-level fitness (Harder and Prusinkiewicz, 2013) and, at the macroevolutionary scale, multiple transitions between distinct gross morphologies have occurred across the angiosperm phylogeny (Stebbins, 1973, 1951). As a result of such a large diversity of complex structures, they are notoriously difficult to place into a meaningful classification scheme (Prenner et al., 2009; Endress, 2010). Here we focus on a form of condensed inflorescence where flowers are closely clustered together, colloquially described as “umbels.” True umbels, according to Troll, Weberling and Endress, are strictly racemose derived structures (Troll, 1937; Weberling, 1992; Endress, 2010). However, umbel-like inflorescence structures, hereafter referred to as *umbellate*, appear in many groups of monocots. The most well-known umbellate structure is the inflorescence of the family Amaryllidaceae *sensu* APG IV (The Angiosperm Phylogeny Group, 2016), which includes *Allium* (onions). The developmental morphology of a wide number of taxa in the family Amaryllidaceae has been investigated, and the morphological consensus is that the inflorescence comprises an indeterminate primary axis with cymose lateral branches (Bravais and Bravais, 1837; Blaauw, 1931; Luyten and van Waveren, 1938; Hartsema and Leupen, 1942; Mann, 1959; Roh et al., 1992; Theron and Jacobs, 1994; Kamenetsky, 1997; Slabbert, 1997; Kodaira and Fukai, 2005; Zhang et al., 2011). The number of lateral cymose branches and the number of flowers per branch can vary across the family, with multiple evolutionary events leading to uniflory (Meerow, 2010). The term thyrse (= raceme of cymes) has been used to describe the inflorescence of some *Allium* (e.g., Kodaira and Fukai, 2005) and can broadly be used to describe the inflorescence in other members of Amaryllidaceae. The only known exception within the family is the non-umbellate *Allium spicatum* (originally described as a separate genus *Milula*, an anagram of *Allium* for its similarities), however the developmental morphology of this endangered species has not yet been investigated (Friesen et al., 2000). Taken together, the inflorescence of Amaryllidaceae is not a true umbel; rather, it represents an example of convergent evolution, arriving at an umbellate form from a non-racemose ancestral structure.

Similar umbellate inflorescences exist in other families of monocots. In particular developmental morphology has been investigated in *Tacca* (Taccaceae: Dioscoreales), at the time used to affiliate *Tacca* with Amaryllidoideae (Eichler, 1879), in *Triteleia laxa* (Asparagaceae: Asparagales) (Han et al., 1994) and in two members of Alismatales *Limnocharis flava* (Alismataceae) and *Butomus umbellatus* (Butomaceae) (Wilder, 1974). While developmental morphological studies provide detailed descriptions of branching, they do not contextualize these patterns within the broader phylogenetic or evolutionary context. Further, inflorescence morphology is almost exclusively assessed *via* developmental morphological investigation of the inflorescence meristem, characterizing the observed sequence of branching events based on emergence of branch, bract and floral primordia. In condensed inflorescences, assessment of branching order is obscured by lack of internodal elongation and can be difficult if not impossible to observe (Wilder, 1974).

**FIGURE 1:**
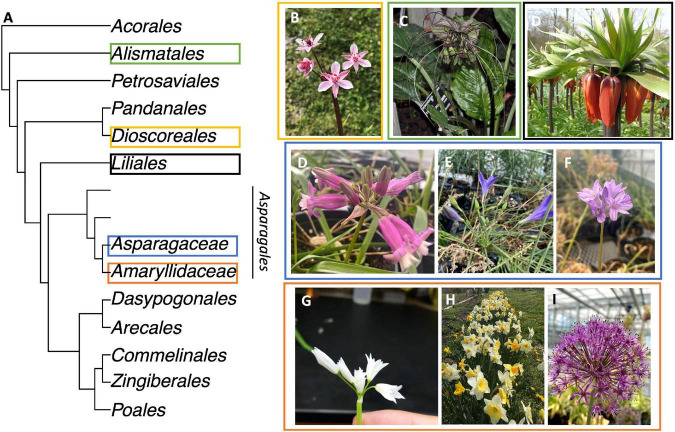
Taxa with Umbellate inflorescence investigated. **(A)** Order level phylogeny of Monocots following (Givnish et al., 2018). The order Asparagales is separated to show two families studied; the unnamed grade represents the remainder of the families in the order. **(B)** *Butomus umbellatus*. **(C)** *Tacca chantrieri*. **(C)** *Fritillaria imperialis*. **(D)** *Dichelostemma* × *venustum* “Pink Diamond”. **(E)** *Triteleia laxa*. **(F)** *Dichelostemma congestum*. **(G)** *Allium triquetrum*. **(H)** *Narcissus “martinette*”. **(I)** *Allium hollandicum*. Colored rectangles correspond between **(A–I)**.

The aim of this study is to determine if the umbellate inflorescence in monocots evolved *via* morphological parallelism or convergent evolution. To test this question, we characterize the developmental morphology of a condensed inflorescence evolved independently in five lineages (Table 1): Taccaceae (Dioscoreales; Figures 1A,B); Butomaceae (Alismatales; Figures 1A,C); and Liliaceae (Liliales; Figures 1A,C), the subfamily Brodiaeoideae (Asparagaceae; Asparagales; Figures 1A,D–F), including a putative reversal to a raceme (Figure 1F); Amaryllidaceae (Asparagales) (Figures 1A,G–I). We revisit the inflorescence of *Triteleia laxa* (Asparagaceae: Asparagales) (Han et al., 1994) and *Butomus umbellatus* (Wilder, 1974; Eckert et al., 2000) (Butomaceae: Alismatales) providing further investigation of the architecture which has previously been obscure. We contrast inflorescence morphologies with racemes found in three members of the subfamily Scilloideae; O*rnithogalum umbellatum, Ornithogalum nutans*, and *Scilla siberica “Alba”* (Asparagaceae; Asparagales), as raceme developmental morphology is consistent through ought monocot (Remizowa et al., 2013). To complement developmental morphological studies, we reconstruct the three dimensional (3D) anatomical vascular branching patterns *via* a serial section generated by laser ablation tomography (LAT) (Strock et al., 2019) of *Butomus umbellatus* and *Ornithogalum umbellatus*. We contextualize our descriptions within broader studies of the developmental morphology of condensed inflorescences in the monocots, including other orders Zingiberales and Commelinales.

**TABLE 1:** Taxonomic and voucher information for taxa in this study.

Taxonomic information	Source	Herbarium voucher
*Butomus umbellatus* L.	Collected, Ithaca, NY	CU JMG001
*Allium triquetrum* L.	Collected, Berkeley, CA	CU JMG002
*Dichelostemma congestum* (Sm.) Kunth	Purchased, Easy to Grow Bulbs, CA	CU JMG003
*Dichelostemma* × *venustum* “Pink Diamond” (Greene) Hoover.	Purchased, Easy to Grow Bulbs, CA	CU JMG004
*Triteleia laxa* Benth.	Purchased, Easy to Grow Bulbs, CA	CU JMG005
O*rnithogalum umbellatum* L.	Purchased, Easy to Grow Bulbs, CA	CU JMG006
*Ornithogalum nutans* L.	Purchased, Easy to Grow Bulbs, CA	CU JMG007
*Narcissus “martinette”*	Purchased, Royal Anthos, Hillegom, The Netherlands	CU JMG008
*Scilla siberica “Alba”* Andrews.	Purchased, Royal Anthos, Hillegom, The Netherlands	CU JMG009
*Allium hollandicum “purple sensation”* R. M. Fritsch.	Purchased, Royal Anthos, Hillegom, The Netherlands	CU JMG0010
*Fritillaria imperialis “rubra maxima”* L.	*Purchased*, *Van Engelen Nursary, CT*	CU JMG0011
*Fritillaria persica* L.	Purchased, Van Engelen Nursary, CT	CU JMG012
*Tacca chantrieri* André.	New York Botanical Gardens	NYBG

## Materials and Methods

### Plant Material and Growth Conditions

Plant were either purchased, collected from the wild or sourced from New York Botanical Gardens (Table 1). All purchased plants were grown at Cornell University Kenneth Post Laboratory facilities. They were potted in Lambert LM-AM potting mix and watered before placing in a cooler at 10°C on Plants were moved from 10 to 4°C on 1/18/2021, and to 1.5°C on 2/6/2021. Potted plants were watered once while in the cold. Plants were transferred to the greenhouse on 05/01/2022.

### Developmental Morphology of Inflorescences

Meristems were stained and imaged with incident light illumination as originally described by Sattler (1968) and later expanded on Posluszny et al. (1980), Charlton et al. (1989), Lacroix and MacIntyre (1995), and Dadpour et al. (2008). Bulbs, corms, or rhizomes were dissected down to pieces of tissue that would fit in a 20 mL scintillation vial. Material was fixed in Formalin Acetic acid Alcohol (FAA: 50% EtOH, 10–37% Formalin, 5% Glacial acetic acid, 35% H_2_O) with vacuum infiltration and stored for at least 24 h. Samples were washed once in 50% EtOH and then taken through a graded EtOH dehydration series (50, 70, 70, 80, 90, 95%) for 1 h at each step. Samples were stained for 1–4 h in a 1% w/v Nigrosin solution (Dadpour et al., 2011) dissolved in 95%EtOH, washed twice in 95% EtOH and further dissected in 95% EtOH to expose the meristem. If the meristem was not sufficiently exposed and stained, we repeated the prior step until meristems had sufficient contrast. Throughout the process FAA and Nigrosin were reused and 95% EtOH was used instead of 100% (Ruzin, 1999). Samples were mounted in 100% room-temperature-vulcanizing silicone (DAP Gasket Maker; Maryland, United States) which remains malleable if submerged in 95% EtOH. We captured image stacks at 5 micrometers, beginning at the apex, using a Leitz Ultropak incident light illuminator microscope (Wetzlar, Germany) using 3.8×, 6.5× or 11× with dipping cone objectives. Large samples were imaged on a Nikon SMV1500 stereo scope (Tokyo Japan). In both cases parafilm was added in the light path if cellular structure caused refraction. Images were captured with a Nikon Digital Sights Fi-3 camera running Nikon Elements F software (version 4.60). We performed focus stacking using the software Picolay (version: 2020-10-27) with 4 filter settings. In cases where an alignment correction was necessary, we used the “2× align” parameter, which aligns first and focuses second. Image post-processing was done in FiJI (version 1.53c): Images were converted to gray scale (i.e., converted to 8-bit), contrast and brightness were adjusted, and a scale bar was added.

### Anatomical Serial Section *via* Laser Ablation Tomography and 3D Reconstruction

Serial anatomical sections were obtained using laser ablation tomography as described in Strock et al. (2019). Plant materials were fixed in FAA and dehydrated in a graded ethanol series then critically point dried. Samples were loaded on the LAT and videos of serial sections were captured at 30 frames/second; the stage advanced at 30 microns/second producing videos that captured 1 micron/frame. Videos were segmented into individual frames with a VLC media player (VideoLAN, France). Xylem tissue in individual frames was manually traced using FIJI and binary masks were generated with the “Mask From ROIs” plugin (Thomas and Trehin, 2021). Composite hyperstacks were generated using the raw stack and masks. 3D images were made with a 3D viewer in FIJI (Schindelin et al., 2012). For *Butomus umbellatus*, we scanned an inflorescence approximately 4 mm with LAT (Supplementary Figure 1A) and rendered the inflorescence in 3D with an image stack composed of serial sections taken every 15 um (Supplementary Files 1, 2). For *Ornithogalum umbellatum*, we scanned an inflorescence approximately 3.6 mm with LAT (Supplementary Figures 1B,C) and rendered the inflorescence in 3D with an image stack composed of serial sections taken every 30 um (Supplementary Files 5, 6).

## Results

**FIGURE 2:**
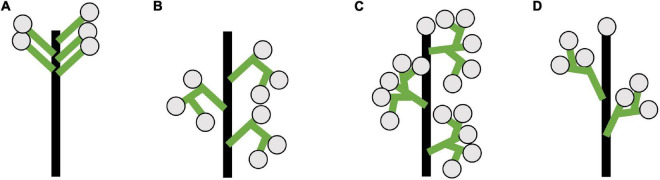
Diagrams of inflorescence architecture observed for umbellate inflorescences in this study. **(A)** Sciadium, a type of true umbel. **(B)** Thyrse with monochasial bostryces. **(C)** Thyrsoid with dichasia. Represents an early developmental stage *Butomus umbellatus* as in later stages cymes become monochasial. **(D)** Thyrsoid with monochasial cincinni. **(B–D)** Depicted without differential internode lengths would be observed in umbellate taxa. Floral subtending bracts (aka pherophylls) and floral bracts (aka prophylls) are not included these depiction (see Prenner et al., 2009: Box 1).

We summarize the main inflorescence architectures observed in the umbellate taxa studied (Figure 2). We observe sciadium (aka indeterminate umbels), a special case of a botryums (aka indeterminate racemes) where there is differential internodal elongation of the primary access with respect to the floral pedicels (Figure 2A). Additionally, we observe three types of thyrses/thyroids (Figures 2B,C). They differed in whether their primary axis terminated in a flower (thyrsoid; Figures 2C,D) or not (thyrse; Figure 2B), and the arrangement of the lateral monochasial cymose branching pattern as either a bostryx (helicoid cymes; Figures 2B,C) or a cincinnus (scorpioid cymes; Figure 2D). These latter differ in their branching site, alternating in left-right in cincinni vs. either left or right in bostryces. Lastly, we observed a thyrsoid with lateral dichasia, cymes that branched twice, in contrast to monochasia cymes that branch once. While there is a classification system for racemose type inflorescence with differential internode length (i.e., umbels, heads, spikes), one does not exist for thyrses/thyrsoids (Endress, 2010). Therefore, these were depicted without differential internode lengths.

### Brodiaeoideae Inflorescence Development

**FIGURE 3:**
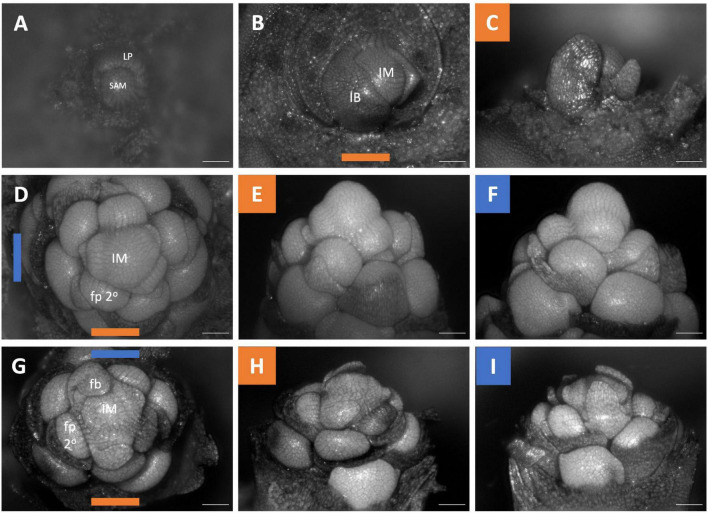
*Dichelostemma congestum* inflorescence developmental morphology. **(A)** Shoot Apical Meristem. **(B,C)** Inflorescence meristem imaged from the apex **(B)** and laterally **(C)**. **(D–F,G–I)** Two stages of inflorescence development, with meristems in the first column **(D,G)** imaged from the apex and the second **(E,H)** and third **(F,I)** columns imaged laterally. Colored lines on images taken from the apex indicate orientation of the inflorescence in subsequent panels [e.g., orange and blue lines in **(D)** represent orientation of images in **(E)** (orange) and F (blue)]. Structures only labeled in first apical image. SAM, shoot apical meristem; LP, leaf primordia; IM, inflorescence meristem; IB, Inflorescence bract; FP, floral primordia; fb, floral bract; 2°–second order flowers. Scale bar of all images: 100 μm.

**FIGURE 4:**
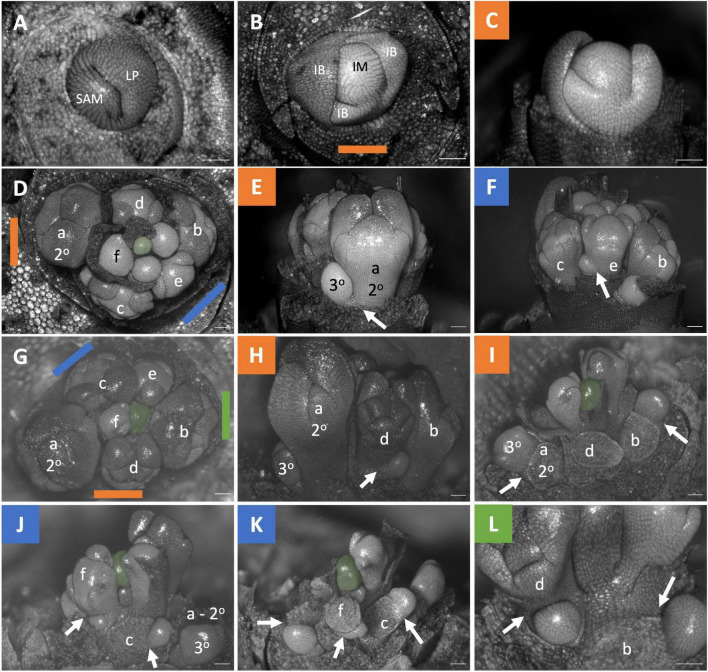
*Dichelostemma* × *venustum* “Pink Diamond” inflorescence developmental morphology. **(A)** Shoot Apical Meristem. **(B,C)** Inflorescence meristem imaged from the apex **(B)** and laterally **(C)**. **(D–F)** Young inflorescence meristem imaged from apex **(D)** and laterally **(E,F)**. **(G–L)** An older stage of inflorescence meristem. Imaged apically **(G)** and laterally **(H–L)**. The first 6 second-order flowers lettered a–f indicating sequence of initiation **(D–L)**. Branching order (2°, 3°) labeled only for the cyme of the first flower initiated a. Colored bars on images taken from the apex **(B,D,G)** indicate orientation of subsequent lateral images. Structures only labeled in first apical image. SAM, shoot apical meristem; LP, leaf primordia; IM, inflorescence meristem, false colored green **(D–L)**; IB, Inflorescence bract; FP, floral primordia; fb, floral bract. White arrows indicate branching events between second and third order branches. Scale bar of all images: 100 μm.

**FIGURE 5:**
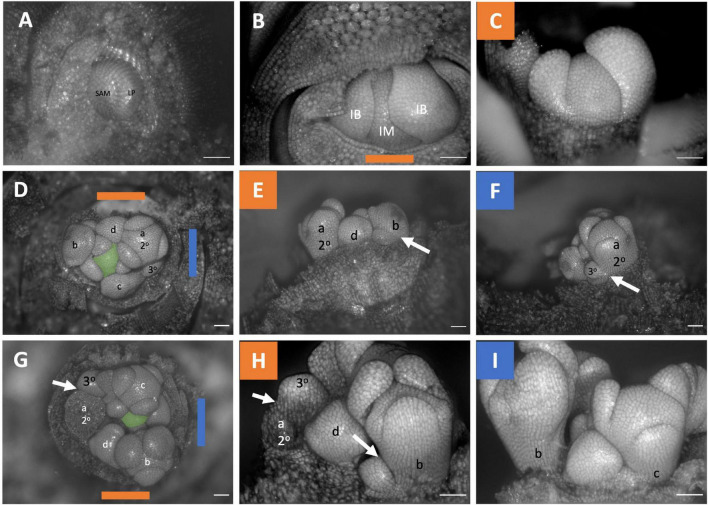
*Triteleia laxa* inflorescence developmental morphology. **(A)** Shoot Apical Meristem. **(B,C)** Inflorescence meristem imaged from the apex **(B)** and laterally **(C)**. **(D–I)** Two stages of inflorescence development. (**D–I)** Two series of inflorescence development. The meristems in the first column **(D,G)** are imaged from the apex and the second **(E,H)** and third **(F,I)** columns are imaged laterally. Colored lines on images taken from the apex **(B,D,G)** indicate orientation of the inflorescence in subsequent panels. The first 4 second-order flowers lettered a-d indicating sequence of initiation **(D–I)**. Branching order (2°, 3°) labeled only for the cyme of the first flower initiated a. SAM, shoot apical meristem; LP, leaf primordia; IM, inflorescence meristem, false colored green **(D,G)**; IB, Inflorescence bract. White arrows indicate branching events between second and third order branches. Scale bar of all images: 100 μm.

The inflorescences of all three examined *Brodiaeoideae* taxa are terminal. After the vegetative shoot apical meristem transitions to the reproductive meristem, the corm dies back, and axillary vegetative meristems develop into individual and separate corms. In all taxa, the vegetative shoot apical meristem is small with distichous phyllotaxy (Figures 3A, 4A, 5A). The transition to the reproductive meristem is marked by an increase in meristem size and the formation of inflorescence bracts (Figures 3B,C, 4B,C, 5B,C). Here the development of *Dichelostemma congestum* deviates from *Dichelostemma* × *venustum* “Pink Diamond” and *Triteleia laxa. Dichelostemma congestum* exhibits internode elongation of the primary axis and lacks secondary branching systems (Figure 3). The inflorescence meristem remains elongated as it produces a series of floral meristems (Figures 3D–F), eventually decreasing in size until no new floral meristem primordia are formed (Figures 3G–H). No prophylls were observed on in this taxon. Development of the inflorescence in *Dichelostemma* × *venustum* and *Triteleia laxa* are similar to one another (Figures 4, 5). In both taxa, the inflorescence meristem gives rise to, in a spiral fashion, multiple second order flower primordia with floral prophylls (Figures 4D, 5D). In the axil of these prophylls arises the tertiary order flower primordia (Figures 4D–L, 5D–I; white arrows). Unlike in *Dichelostemma congestum*, there is little internode elongation of the primary axis (Figure 4L: Flower d is positioned higher than flower b). As such, secondary flower primordia are horizontally adjacent to the tertiary flower primordia (Figures 4E, 5H). Taken together the inflorescence of *Dichelostemma congestum* can be considered a raceme (Figure 2A) and those of *Dichelostemma* × *venustum* and *Triteleia laxa* can be considered a thyrse (Figure 2B), i.e., a primary raceme axis with multiple secondary branches exhibiting a cymose branching pattern specifically, a bostryx (aka helicoid cyme).

### Amaryllidaceae Inflorescences Development

**FIGURE 6:**
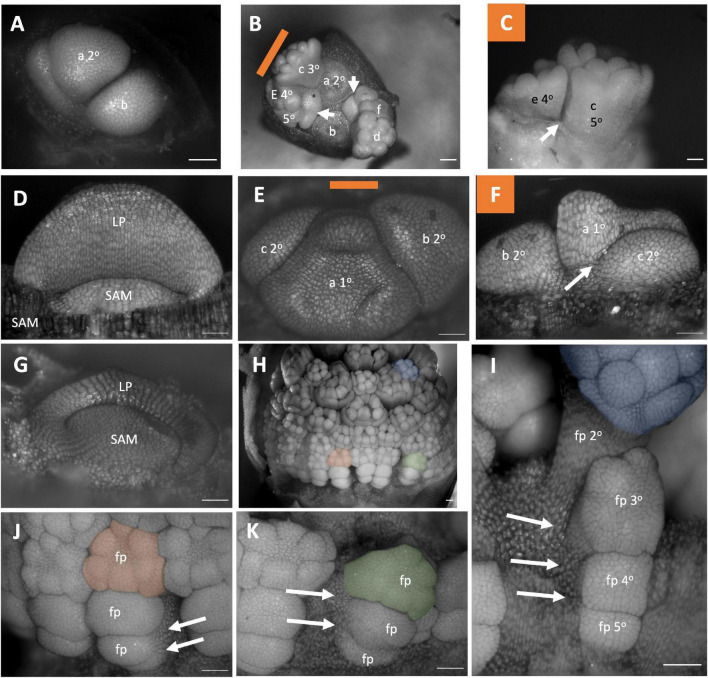
Amaryllidaceae Inflorescence developmental morphology. **(A–C)** *Allium triquetrum* **(A)** Young inflorescence with first two flower primordia. **(B,C)** Older inflorescence, with the first two flower dissected away, imaged from the apex **(B)** and laterally **(C)**. **(D–F)** *Narcissus “martinette”* shoot apical meristem **(D)** and young inflorescence **(E,F)**. Colored lines on images taken from the apex indicate orientation of the inflorescence in subsequent panels. The first flowers **(A–C,E,F)** lettered a–f indicating sequence of initiation. Branching order (1°, 2°, 3°) labeled only for the cyme of the first flower initiated). **(G–K)** *Allium hollandicum.* **(G)** shoot apical meristem. Young inflorescence **(H)** and close-up of three flower primordia **(I–K)** false colored for orientation. Branching ordered specified in **(I)**. Colored lines on images taken from the apex **(B,E)** indicate orientation of the inflorescence in subsequent panels. SAM, shoot apical meristem; LP, leaf primordia; fp, floral primordia. White arrows indicate branching events. Scale bar for all images: 100 μm.

In *Allium triquetrum* the primary axis is indeterminate, and the inflorescence meristem branches twice, producing two secondary flowers which continue to branch in a cymose pattern (Figures 6A,B: Flowers a and b). The pedicels of flowers within the same branching system appear horizontally adjacent to each other (Figure 6C: Flowers e and c). The inflorescence of *Narcissus “martinette”* contains three to four flowers but only three flower primordia were observed here (Figures 6E,F). It is a determinate inflorescence with two lateral flowers (Figure 6F). In both *Allium triquetrum* and *Narcissus*, flower pedicels appear horizontally adjacent to each other (Figures 6C,F). A*llium hollandicum* exhibits a different adult inflorescence morphology. This taxon experiences internodal elongation of the primary axis (Figure 6H), with the presence of secondary and higher order branching similar to that seen in *Allium triquetrum* (Figures 6I–K). Due to the internodal elongation of the primary axis, all flower pedicels are vertically adjacent to each other (Figures 6G–F). All three species lack both floral subtending bracts (pherophylls) and floral bracts (prophylls). The inflorescence of *Allium triquetrum* is a thyrse with two lateral bostryx (Figure 2B). The *Narcissus* inflorescence observed here be considered a sciadioid (determinate umbel). *Allium hollandicum* inflorescence is interpreted as a thyrse, as previously described for other ornamental *Alliums* (Kodaira and Fukai, 2005).

### *Butomus umbellatus* Inflorescence Development

**FIGURE 7:**
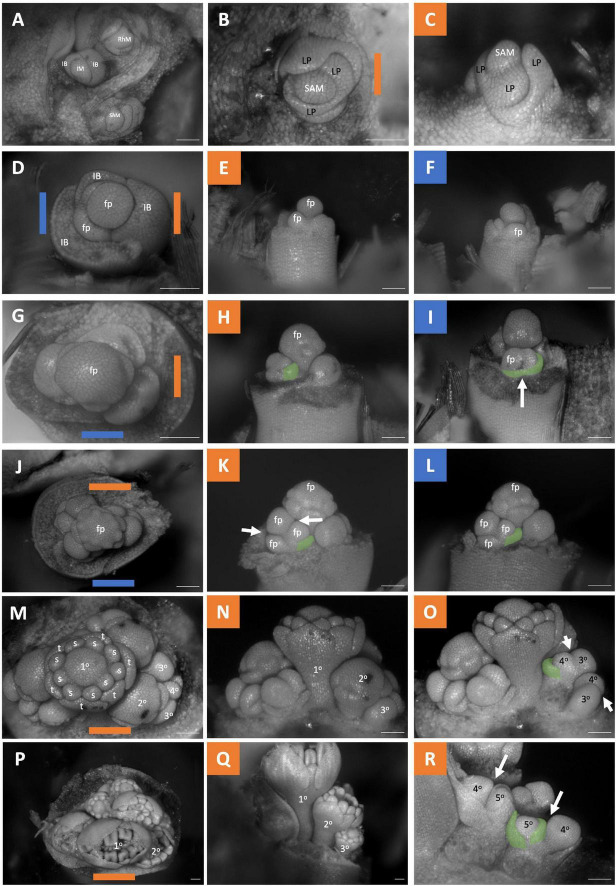
*Butomus umbellatus* inflorescence developmental morphology. **(A)** Leading end of rhizome including three meristems. Shoot Apical Meristem, Inflorescence Meristem, Rhizome meristem. **(B,C)** SAM, **(B)** SAM imaged from the apex. **(C)** SAM imaged laterally. **(D–R)** Five stages of inflorescence development (rows). The meristems in the first column **(D–P)** are imaged from the apex. The second and third columns are imaged laterally. **(D–F)** Inflorescence with three inflorescence bracts formed, the oldest inflorescence bract (left) removed, and the second oldest inflorescence bract (right) removed in **(E)**. **(G–I)** Inflorescence where the first whorl of tepal primordia arise. **(J–L)** Apical flower with nine stamen primordia. **(M–O)** Inflorescence with nine stamen primordia fully formed, gynoecium developmental about to initiate. **(P–R)** Inflorescence with gynoecium development. Branching order (1°–5°) labeled for one of the three cymes **(M–R)**. Colored lines on images taken from the apex **(B–P)** indicate orientation of the inflorescence in subsequent panels. SAM, shoot apical meristem; IM, inflorescence meristem; RhM, lateral rhizome meristem; LP, leaf primordia; IB, Inflorescence bract; fb, floral bract, false colored green **(H–I,K–L,O,R)** fp, floral primordia; t, tepal primordia; s, stamen primordia. White arrows indicate branching events. Scale bar for all images: 100 μm.

The development morphology of the *Butomus umbellatus* inflorescences has been described in detail elsewhere (Wilder, 1974; Eckert et al., 2000); however, prior studies lacked all developmental stages and key questions remain concerning the architecture (Figure 7). The inflorescence meristem produces three bracts, each with an axillary secondary bud (aka primary bud *sensu* Wilder) that in turn gives rise to a secondary lateral branch that terminates into a flower (Figures 7A,D–F). The primary axis terminates in a flower While it appears that buds arise simultaneously because as all buds are approximately at the same developmental stage at any moment in time, they arise sequentially following their respective bract origin (Figures 6A,D–F). Following these secondary branching events, the primary axis transitions into a floral meristem (Figures 7D,G,J,M,P). We find that each secondary branch branches sequentially twice as opposed to a trifurcation (Figures 7I,K) before the secondary branch meristem terminates into a flower (the three flowers on the secondary lateral branch are collectively referred to as triads *sensu* Wilder). Secondary branches are displaced from the primary stem axis appearing horizontal adjacent to the primary axis, as opposed to branching from the axis (Figures 7K,N). This branching pattern is repeated in subsequent order branching events (Figures 7M–R). However, in latter orders of branching, there may be more than two branching events as evident by meristem primordia that are not divisible by three. Therefore, the inflorescence can be considered a thyrsoid with three lateral dichasia that switch to monochasia, specifically bostryces, later in development (Figure 2C).

### *Fritillaria* Inflorescences Development

**FIGURE 8:**
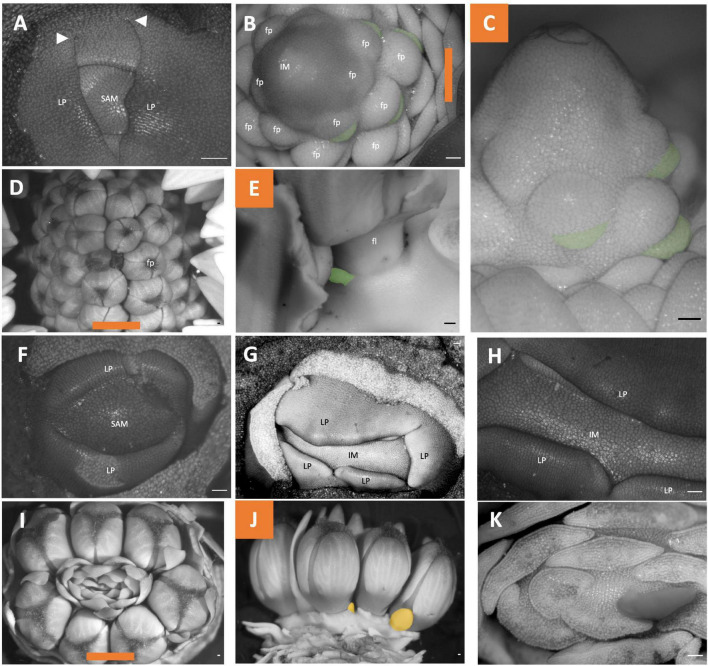
*Fritillaria* inflorescence developmental morphology. **(A–E)** *Fritillaria persica.* **(F–K)** *Fritillaria imperialis.* **(A)** SAM. **(B–C)** Young inflorescence meristem with floral primordia. **(B)** imaged apically **(C)**, Imaged laterally. **(D,E)** Mature inflorescence **(D)** imaged apically **(E)** imaged laterally following dissection. **(F)** SAM. **(G–H)** Young inflorescence meristem exhibiting fasciation. **(I–K)** mature inflorescence **(I)** imaged apically **(J)** Imaged laterally following dissection of floral subtending bracts **(K)** Imaged apically following dissection of leaves. Colored lines on images taken from the apex **(B,D,I)** indicate orientation of the inflorescence in subsequent panels. Floral subtending bracts are false colored green **(B,C,E)**, floral bracts false colored orange **(J)**. White arrowheads indicate leaf fusion. SAM, shoot apical meristem; IM, inflorescence meristem; fp, floral primordia; LP, leaf primordia. Scale bar of all images: 100 μm.

*Fritillaria persica* and *Fritillaria imperialis* exhibit distinct inflorescence structures. The inflorescence meristem of *Fritillaria persica* initiates as a large swelling. It initiates leaf primordia that whose proximal margins are congenitally fused (Supplementary Figure 2 and Figure 8A: white arrowheads). The inflorescence initiates floral meristems in a spiral phyllotaxy with flower subtending bracts; however, the bract does not cover the floral primordia (Figures 8B,C: false colored green). As a result, the floral subtending bract never fully develops and remains a rudimentary organ present in more mature inflorescences (Figure 8E: false colored green). Floral bracts are not observed in this taxon. The inflorescence is indeterminate, continuing to produce flowers for as long as the meristem functions (Figure 8D). In contrast the shape of the inflorescence meristem of *Fritillaria imperialis* is fasciated (an oval shape; Figures 8F–H). While the meristem is fasciate the mature inflorescence stem is round, due to shape imprinting (Endress, 2008; Figure 1A). Organ primordia are initiated spirally with little internodal elongation between organs (Figure 8I). Flowers initiate sequentially, but development is synchronized, and all flower pedicels end up adjacent to each other (Figure 8J). The meristem is indeterminate continuing to produce leaf primordia indefinitely (Figure 8K). The flowers of *Fritillaria imperialis* also have rudimentary floral bracts (Figure 8J: false colored orange). The inflorescence of *Fritillaria imperialis* can be considered a botryum (indeterminate raceme) while that of *Fritillaria persica* can be considered a sciadium (Figure 2A).

### *Tacca chantrieri* Inflorescences Development

**FIGURE 9:**
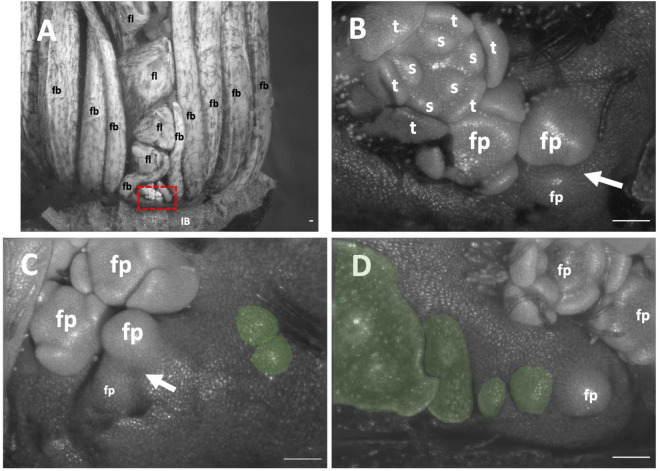
*Tacca chantrieri* inflorescence developmental morphology. **(A)** One of the two lateral cincinni on an inflorescence. **(B)** A zoom-in of red box in panel A. **(C)** Cincinnus with a branching event and two newly formed bract primordia (false colored green). **(D)** Cincinnus that is not currently branching, with two floral bract primordia, the two oldest bracts have been dissected but scar is false colored green. White arrows indicate branching events. IB, Inflorescence bract; fb, floral bract, false colored green **(C,D)** fp, floral primordia; fl flower; t, tepal primordia; s, stamen primordia. White arrows indicate branching events. Scale bar of all images: 100 μm.

Only unopened inflorescences that had emerged from the stem were available. The inflorescence was interpreted as comprising two cincinni (aka scorpioid cymes), surrounded by four inflorescence bracts, each branching centrifugally along the transverse plane (Figures 9A,B). Flowers did appear to not directly formed in the axils of their floral bracts (Figures 9C,D green); floral bracts development was delayed appearing in two rows sides of the inflorescences flanking the flowers (Figure 9A). Similar to other cases, pedicels of all flowers were horizontally adjacent due to displacement of lateral flower primordia from their branch. Taken together, this inflorescence is classified as a thyrsoid with two lateral cincinni (Figure 2D).

### Scilloideae Inflorescence Development

**FIGURE 10:**
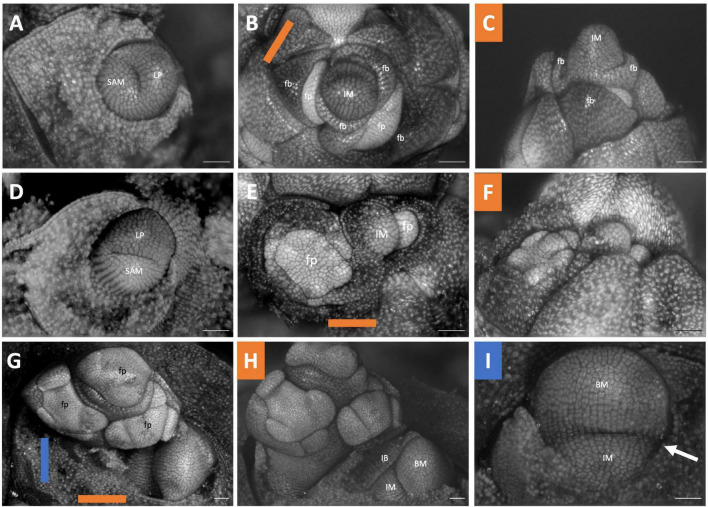
Scilloideae inflorescence developmental morphology. **(A–C)** *Ornithogalum nutans* **(D–F)** *Ornithogalum umbellatum* **(G–F)** *Scilla siberica* “*Alba*”. **(A)** Shoot Apical meristem. **(B,C)** young inflorescence imaged **(B)** apically **(C)** laterally. **(D)** Shoot Apical meristem **(E,F)** young inflorescence imaged **(E)** apically **(F)** laterally. **(G)** Young inflorescence imaged apically. **(H)** Imaged laterall**y** showing a racemic pattern of development of a branch **(I)** Inflorescence meristem with branching events. Colored lines on images taken from the apex **(B,E,G)** indicate orientation of the inflorescence in subsequent panels. SAM, shoot apical meristem; LP, leaf primordia; IM, inflorescence meristem; fp, floral primordia; fb, floral bract; IB, inflorescence bract; BM, branch meristem. Scale bar of all images: 100 μm.

The inflorescence development of three members of the Scilloideae, all of which represent classic racemes, were studied. The development of *Ornithogalum nutans* (Figures 10A–C) and *Ornithogalum umbellatum* (Figures 10D–F) parallel each other. Each represents simple racemes where flowers are arranged in a spiral phyllotaxy. The inflorescence does not terminate in a flower (Figures 10C,F). The inflorescence of *Scilla siberica* is similar; however, this taxon exhibits double racemes (Figures 10G–I). The inflorescence meristem branches twice or more to produce racemes each of which contains three to five flowers (Figure 10H). The primary axis lacks any internodal elongation, only the lateral branches elongate and emerge above the soil. *Ornithogalum umbellatum* as a representative of the raceme condition (Supplementary Figures 1B,C). From the LAT scan of this inflorescence, we extracted serial sections every 30 μm of distance along the inflorescence and rendered the inflorescence manually traced two vasculature strands from the apex toward the basis (Supplementary Files 5, 6).

### Inflorescence Vascular Anatomy

To obtain complementary evidence of branching order, we traced vascular bundles in serial sections of *Butomus umbellatus*, as a representative of the umbellate inflorescence, and *Ornithogalum umbellatum* as a representative of a raceme inflorescence. We used LAT to obtain the fine scale serial sections where contrast between anatomical features is generated by differential autofluorescent signature of the cell wall under UV excitation.

**FIGURE 11:**
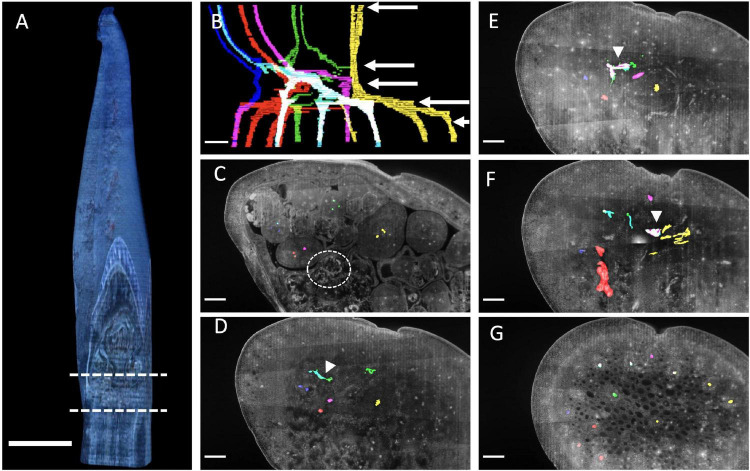
*Butomus umbellatus* inflorescence anatomy. **(A)** Inflorescence reconstructed from serial sections at 15 μm increments. Region in between dashed lines correspond to the section of vasculature in **(B)**. **(B)** Outlined vascular bundles in the absence of other tissue. Colors correspond to different flowers (1) yellow, (2) red and magenta, (3) green, (4) blue and cyan. White arrow corresponds to the individual section, in descending order, shown in the following panels. **(C–G)** Individual anatomical panels, in grayscale. Circle indicates meristematic tissue. White arrowheads indicate when vascular bundles from distinct flower pedicels merge. Scale bar of all images: 500 μm.

Within the 4mm sample of *Butomus umbellatus* (Figure 11A) we focused our analysis on a 1.25 mm region where the inflorescence stem meets floral pedicels (Figure 11A dashed lines B; Supplementary Files 3, 4). Vascular bundles could be manually discriminated relative to surrounding tissue by a distinct autofluorescent signature generated by differences in the secondary cell wall composition of these bundles relative to the surrounding ground tissue. We will refer to vascular bundles as merging if two bundles come together relative to their acropetal position in the flower. Moving from the flower pedicels to the inflorescence stem, floral pedicels are composed of three vascular bundles (Figure 11D). Two of the three pedicel vascular bundles always merged (red puncta in Figure 11D merge by Figure 11E, and blue puncta in Figures 11D,E merge by Figure 11F), which resulted in two vascular bundles at the base of each flower pedicel. Of these two bundles, one bundle always merges with the vascular structure associated with the lateral axis from which it branched (Figures 11D–F: white arrowhead). We interpret this as anatomical evidence of branching. The other bundle arises from an independent vascular bundle found within the inflorescence stem. Each flower, except the terminal flower on the primary axis, is subtended by a bract. We were only able to trace the vascular bundle of the bract subtending the secondary branch. The primary vascular bundle initiates from a single vascular bundle in the inflorescence stem.

**FIGURE 12:**
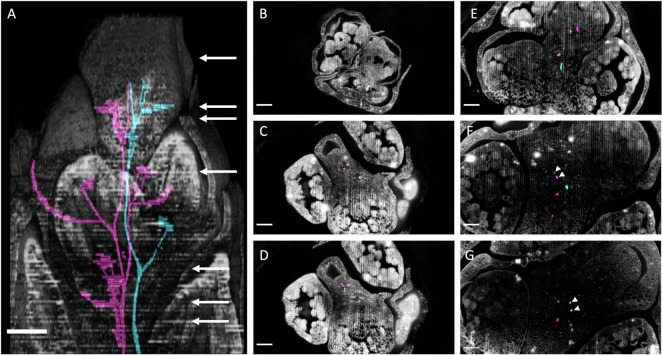
*Ornithogalum umbellatum* inflorescence anatomy by laser ablation tomography. **(A)** Inflorescence reconstructed from serial sections at 30 um increments overlayed with two vascular bundles (cyan and magenta). White arrow corresponds to the individual section, in descending order, shown in the following panels. **(B–G)** Individual anatomical panels, in grayscale. White arrowheads indicate when vascular bundles from distinct flower pedicel merge. Scale bar of all images: 250 μm.

In *Ornithogalum umbellatum* we manually traced two vasculature strands from the apex toward the basis (Figure 12A; Supplementary Files 5, 6). Like *Butomus umbellatus* the flower pedicels had three vascular strands (Figures 12C,D: magenta). Vascular strands both split into two strands (Figures 12E,F magenta; Figures 12F,G cyan). Stem twisting was expressed in the vascular strands, they rotated along the inflorescence stem due to branching.

We attempted LAT scans on the young meristematic tissue of *Allium hollandicum* (Supplementary File 7) but due to the delicate, under-developed cell wall structure of this tissue, this sample was difficult to visualize and experienced “burning” under UV excitation. Similar difficulties in visualizing the anatomy of delicate samples with LAT were observed within *B. umbellatus*, however this “burning” was localized to young meristematic tissue (Figure 11C: dashed circle). Because LAT is primarily utilized for the visualization of plant cell walls, this technology is ideally suited for tissue-level visualization of larger samples with rigid cell wall structure.

## Discussion

Here we present a developmental morphological and anatomical investigation into umbellate inflorescence structures across the monocots. We use a novel approach to characterize the notoriously complex vascular system in monocots, focusing on the inflorescence (Zimmermann and Tomlinson, 1972; Vita et al., 2019). Despite the vast evolutionary time scales represented in our sampling (Figure 1), we can identify at least three cases of homoplasy *via* parallelism and at least two cases of homoplasy *via* convergent developmental programs that each result in an umbellate inflorescence. The parallel evolution cases of inflorescence evolution are underlined by a new variant of metatopic displacement termed horizontal concaulesence.

### Developmental Parallelism and Convergence Underlie Umbellate Evolution in Monocots

Using a phylogenetic framework, we have identified nested cases of homoplasy in inflorescence development across the monocots. We identify at least three independent ways to make an umbellate inflorescence: (1) The bostryx- (aka helicoid cyme) derived umbellate inflorescence, (2) The cincinnus- (aka scorpioid cyme) derived umbellate inflorescence, and (3) the racemose-derived umbellate inflorescence which is considered a true umbel (Troll, 1937; Weberling, 1992; Endress, 2010).

Among the bostryx derived inflorescences, there are at least three cases of parallel evolution exemplified by (a) Brodiaeoideae, excluding *Dichelostemma congestum*, (b) the inflorescence of Amaryllidaceae, including *Allium triquetrum*, studied here, and (c) the inflorescence of *Butomus umbellatus*. They share a generative program of inflorescence development involving multiple bostryces (aka helicoid cymes) that lack vertical internodal elongation of the primary axis (Figure 2B). Within this general structure there is large variation, including whether the primary axis terminates in a flower (e.g., *Butomus umbellatus*) or not (e.g., Brodiaeoideae and Amaryllidaceae), and how many bostryces are present in the inflorescence (e.g., *Allium triquetrum* = *2, Butomus umbellatus* = 3). This parallel evolution likely applies to other umbellate taxa that fall in these groups, specifically, *Limnocharis flava* (Alistimataceae Alismatales) which has been described as a cincinnus but diagrammatically resembles a bostryx (Wilder, 1974) and various member of the Amaryllidaceae (Blaauw, 1931; Mann, 1959). Even though the specimen of *Narcissus* observed here is classified as a botryoid, cymose branching likely occurs in individuals with four flowers similar to other members of this family. On the other hand, *Allium hollandicum* does exhibit internodal elongation of the primary axis and lateral cymes arranged differentially than *Allium triquetrum*; this appears to be a derived condition. Further phylogenetic and developmental studies are necessary to assess these inflorescence types.

The inflorescence of *Tacca chantrieri* is a convergent case of umbellate inflorescence originating a different type of thyrse those with cincinni. A cincinnus and a bostryx, both cymes, differ in their three-dimensional floral arrangements (Buys and Hilger, 2003), but are topologically related; hypothetically they can transition between each other through two intermediate inflorescences, rhipidiums and drepaniums (Eichler, 1875; Müller-Doblies, 1977; Müller-Doblies et al., 1992; Weberling, 1992; Hrabovský, 2019). Collectively these four inflorescence types are referred to as monochasia (Eichler, 1879). While, it may have been possible that the lateral cymes in the *Tacca chantrieri* inflorescence were ancestrally derived from a bostryces which transitioned to a cincinni through a rhapidum or drapanium, as have been described in the Amaryllidaceae genera *Clivia* and *Lapideria* (Müller-Doblies and Müller-Doblies, 1978), it is more likely that the ancestor condition was a thyrsoid with uncondensed internodes; a common condition in this order (Remizowa et al., 2010; Nuraliev et al., 2021; Yudina et al., 2022). As such, the this represents a convergent case of an umbellate inflorescence since the lateral cymes are arrange differently than the bostryx derived umbellate taxa. The cincinnus of *Tacca* resemble those found in various member of Commelinaceae (Hardy et al., 2000; Hardy and Stevenson, 2000a,b) specifically *Plowmanianthus* which is interpreted as a thyrse that has reduced to a single cincinnus (Hardy et al., 2004). However, the inflorescence of these taxa are not considered umbellate, exemplifying how similar inflorescence architecture can give rise to diverse overall form.

In contrast to thyrse derived umbellate inflorescences, *Fritillaria imperialis* and *Dichelostemma congestum* have independently evolved a raceme-derived umbellate inflorescence, specifically sciadium and as such they have converged upon a true umbel. In *Fritillaria imperialis*, lack of internodal elongation is likely related to the fasciated meristem found in this taxon (Figures 8G,H). Umbellate inflorescences are not common in this group; most members either have solitary flowers or have a raceme (Beetle, 1944; Rønsted et al., 2005; Huang et al., 2018). Depictions of strongly fasciated stems, compared to the round stems found here (Figure 1D), date back to the 1600s (Basilius, 1613) and as such may have been influenced by artificial selection.

The inflorescence of *Dichelostemma congestum* may have experienced a different evolutionary force —developmental system drift (DSD) (True and Haag, 2001). DSD is defined when two species with a similar overall phenotype, in this case an umbellate inflorescence, evolved from ancestors with different developmental pathways, in this case different inflorescence architectures (i.e., bostryx derived, cincinnus derived and raceme derived). All members of Brodiaeoideae have an umbellate inflorescence (Pires et al., 2001), however, of the species described, only the inflorescence of *Dichelostemma congestum* is raceme-derived. The underlying developmental basis of the inflorescence (i.e., raceme vs. thyrse/thyrsoid), which is expressed in the order in which flowers open, has been noted in taxonomic studies of *Dichelostemma* (Keator, 1967). Order of flower anthesis is an important mechanism that plants use to avoid selfing and promote outcrossing (Harder et al., 2004). Umbellate inflorescences often exhibit synchronous dichogamy, in which all flowers in the umbel go through simultaneous phases of pollen productivity or ovule maturity. This contrasts with acropetal flower maturation, characteristic of raceme inflorescences (Ida and Minato, 2020). In *Dichelostemma*, DSD may be a mechanism whereby the gross flower display of an umbellate inflorescence is maintained, perhaps to attract pollinators, while allowing for changes in the order of flowering time and reproductive success. Field observations are consistent with this; member of the *Dichelostemma* genus have a similar pollination mode, mainly butterfly, except for *Dichelostemma ida-maia* which is likely hummingbird pollinated due to its distinct floral modification with respect to the rest of the genus (e.g., flower is red, no landing pad; Keator, 1967). A similar case may apply to the cincinnus vs. bostryx derived umbels in Amaryllidaceae described above. Further ecological work is needed to test this hypothesis.

### Anatomical Evidence of Branching

Because assessing branching order in a condensed structure can be difficult, as meristematic primordia rise quickly and within a small space, we sought to obtain complementary evidence of branching from anatomical serial sections as has been done before (Abbe, 1935; Maze, 1977). In a the similarly condensed inflorescence of *Musa*, the lateral branching system (i.e., the banana “hand”) is interpreted to be a cincinni based on the order of initiation of the individual flower primordia (Fahn, 1953) but, in *Musa accuminata*, the second flower primordium is vascularized before the first flower primordium, which is inconsistent with expectations for a cincinnus. Later, developmental morphological work shows that branching order within non-crop members of the genus *Musa* exhibits intraspecies variation in order of flowering within a “hand” (Kirchoff, 2017).

Here, the anatomical section from LAT work provided complementary evidence of branching. In the original description of the *Butomus umbellatus* inflorescence, key developmental morphological stages were not observed, leading to an uncertain conclusion as to the branching order within the condensed structure (Wilder, 1974: Figures 60, 62). Our dissections reveal a clear order of primordia emergence during early inflorescence development, indicating a pattern reflecting cymose branching (Figures 7I,K,R). Our results show that the *Butomus* flower pedicel has three vascular strands, two of which merge into a single strand (Figure 11C). A similar pattern has been observed in other Monocots including *Tofieldia* (Alismatales), *Metanarthecium* (Dioscoreales) and *Japonolirion* (Petrosaviales) (Remizowa et al., 2006, 2008, 2013), and is thought to be correlated with the small size of the pedicel. In *Butomus*, one of these pedicel vascular strands immediately originates from the corresponding lateral axis from which it branches (Figure 11 white arrowheads). Interestingly, the other vascular bundle is not derived from the lateral branch but appears to form from tissue located elsewhere within the inflorescence stem. While only one of the two vascular bundles is derived from the lateral branch, we nonetheless interpret this as anatomical evidence of branching; we can consistently trace branching from the quaternary branch in the cyme to the terminal flower of the primary branch. It would be interesting to compare the vascular dynamics of non-umbellate inflorescences with a cyme such as *Thismia* (Nuraliev et al., 2021).

Our anatomical results were obtain using LAT, a new method to obtain anatomical serial section to complement the anatomists’ toolkit. It’s major advantages over traditional approaches to obtain serial sections (i.e., embedding), is speed and scalability. Serial sections of 1um increments can consistently be obtain within a few minutes. This consistency and large amount of data facilitates computational 3D reconstruction as there is no need to interpolate between section. Although, computational 3D reconstruction, can be done with serial section obtain by traditional sectioning (Haushahn et al., 2014). The main disadvantage is this method solely relies on diffraction from plant cell wall limiting for imagine, as such we have found that good resolution is obtained only mature cells. In contrast, the rich history of staining schedules allow anatomist to visual distinct structures cross mature and young issues.

### Umbellate Inflorescence and Horizontal Concaulescence

**FIGURE 13:**
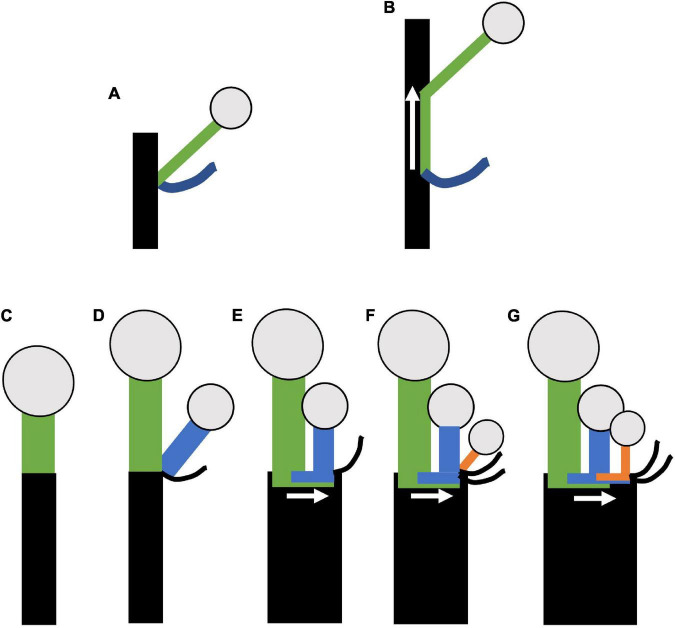
Model of horizontal concaulescence. **(A,B)** Concaulescence following Weberling (1992) **(A)** Typical lateral (axillary) branching. **(B)** Concaulescence; lateral branch is congenitally fused (note overlap of green with black) to the main stem and displaced vertically relative to the subtending bract. **(C–G)** Horizontal concaulescence exemplified in a single cymose branch with two sequential branching events **(D,F)**. **(C)** Stem with terminal flower, inflorescence bract not shown. **(D)** Lateral (axillary) branching event (blue). **(E)** Secondary displacement of the lateral branch (blue) such that the pedicel is horizontally adjacent to the terminal flower. **(F)** A second branching event (orange). **(G)** Secondary displacement of the lateral branch as in **(E)**. Rectangles = stems; Circles = terminal flowers; black curved lines = bracts; arrow = direction of concaulescent growth.

Umbellate inflorescences are often described as lacking internodal elongation. Here we observe that floral pedicels are horizontally adjacent, arising out of flat stem, and often displaced from the pedicel which they branched. Lack of internodal elongation, on its own, is not sufficient explain this form of bud displacement. Instead the organization of thyrse derived umbellate inflorescence structure can be best characterized as a distinct form of metatopy—where buds initiated in an axillary position are displaced secondarily as a result of differential growth (Troll, 1957; Weberling, 1992; Endress, 2010; Kaplan, 2022). Two general forms of metatopy exist: concaulescence, where buds are displaced upwards with respect to the subtending bract (Figures 13A,B) and recaulescence (aka epiphylly), where buds are displaced on the leaf. The developmental basis of the displacement is congenital fusion of the lateral axis to either the primary axis (concaulescence; Figure 13B) or the leaf (recaulescence). Gross morphological outcomes of concaulescence typically result with flowers appearing on the underside of bracts (e.g., *Streptopus amplexifolius, Symphytum officinale, Tricyrtis hybrida)* (Troll, 1937; Fukai, 2009; Kotelnikova, 2011), but can also cause the disassociation between the flower and its glume in a Cyperoideae spikelet (Vrijdaghs et al., 2010) and the adnation of the inflorescence stem to the vegetative internodes in some rattan palms (Fisher and Dransfield, 1977). In these cases, and all other described cases, to our knowledge, metatopy occurs vertically, because the primary axis elongates orthotropically.

We argue that compound inflorescence here exhibits a form of concaulescence occurring plagiotropically which we term horizontal concaulescence (Figures 13C–G). Here the floral buds are displaced horizontally with respect to their subtending bracts when present (Figure 13G). This displacement is due to secondary horizontal cellular elongation of the primary axis (Figures 13E,G). The result is flower pedicels emerging horizontally adjacent to one another, seemingly arising out of a “flat stem.” In all thyrse/thyrsoid umbellate taxa, lateral floral meristematic primordia, are physically attached to the pedicel they branch from and the flat inflorescence stem surface (*Dichelostemma venustum* Figure 4E; *Triteleia laxa* (Figure 5I); *Allium triquetrum* (Figure 6B); *Narcissus* (Figure 7F), *Butomus umbellatus* (Figure 7K), and *Tacca* (Figure 9C). Parallel organization of floral pedicels also occurs in raceme derived *Fritillaria imperialis* (Figure 8J) but there is no observed fusion, instead the large size of the fasciated meristem naturally allows for more spacing. A putative line of evidence for this congential fusion is found in a naturally occurring aberrant inflorescence phenotype in *Agapanthus* sp. (Amaryllidaceae) (Supplementary Figure 3). The typical inflorescence phenotype is similar to others in the family, but the aberrant phenotype exhibits an additional umbel (Supplementary Figures 3A,B). Interestingly, a portion of the inflorescence stem in between the umbels shows a scar (Supplementary Figure 3C: white bracket). We interpret these scars as a morphological marker of congenital fusion between a cymose axis and the inflorescence axis. However, instead of horizontal elongation, as is typical, the inflorescence axis had not finished elongating vertically. Further developmental morphological analysis of the inflorescence will be necessary to understand the development phenotype.

We describe horizontal concaulescence in all non-raceme derived umbellate taxa studied, including *Tacca*, with one exception, the inflorescence of *Allium hollandicum* (Figures 6H–J) which, shares its inflorescence architecture with *Allium rosenbachianum* previously described (Kodaira and Fukai, 2005). In these cases, the primary axis is elongated vertically resulting in a classic case of concaulescence. A similar situation may occur in *Allium spicatum*, that has been described as a raceme, but more detailed morphological studies are necessary to confirm this hypothesis (Friesen et al., 2000). We note that the definition of metatopy may not fully apply to cases where floral bracts have been lost (e.g., members Amaryllidaceae; Figure 5), as no displacement occurs *per se*. However, loss of floral bracts is likely secondary to umbellate evolution and floral pedicels still appear horizontally adjacent to each resulting in a flat stem. Similarly, other monocots within cymose lateral inflorescences, including members of Zingiberales and Commelinales, exhibit a similar flat stem morphology whereby floral primordia arise out of a stem cushion (Kirchoff, 1998, 1997, 1986, 2017; Hardy and Stevenson, 2000a,b; Fukai and Udomdee, 2005; Kirchoff et al., 2009, 2020). This hints at a widespread role of metatopy in the development and evolution of diverse inflorescence architectures in the monocots.

## Conclusion

Understanding the generative development program that gives rise to complex structure is a key step to fully understanding its evolutionary significance. Recent years have seen a resurgence in the use of plant morphology to inform phylogenetic relationships among plants and investigate developmental processes leading to observed patterns of diversification (Bucksch et al., 2017). Here we describe a wide range of developmental morphological variation found in convergent inflorescence phenotypes. Insights from this descriptive investigation identify a previously undescribed plant morphological process. Further these results can provide a source of data for future evolutionary and ecological studies focusing on the form and function of this adaptive plant architecture.

## Author’s Note

The authors dedicate this paper to the memory of TA, who passed away suddenly in December 2021. Tara was a brilliant botanist with a bright future in academia. The findings in this manuscript were expedited by her creative problem-solving and ingenuity. To learn more about Tara and her work visit https://taraatluri.org/.

## Data Availability Statement

The datasets presented in this study can be found in online repositories. The names of the repository/repositories and accession number(s) can be found below: https://doi.org/10.5281/zenodo.6012529.

## Author Contributions

JM-G designed, executed experiments (excluding laser ablation tomography), and wrote the manuscript. TA, IR, and AH contributed to morphological preparation and imaging. CS performed laser ablation tomography. WM provided coolers and growth conditions for plants. All authors edited the manuscript.

## Conflict of Interest

The authors declare that the research was conducted in the absence of any commercial or financial relationships that could be construed as a potential conflict of interest. The handling editor declared a past co-authorship with one of the author DS.

## Publisher’s Note

All claims expressed in this article are solely those of the authors and do not necessarily represent those of their affiliated organizations, or those of the publisher, the editors and the reviewers. Any product that may be evaluated in this article, or claim that may be made by its manufacturer, is not guaranteed or endorsed by the publisher.
