# Supplementary material for: Developmental Morphology and Anatomy Shed Light on Both Parallel and Convergent Evolution of the Umbellate Inflorescence in Monocots, Underlain by a New Variant of Metatopy
Source: Front Plant Sci. 2022 Apr 29;13:873505. doi: 10.3389/fpls.2022.873505 (PMC9100582; doi:10.3389/fpls.2022.873505)
Supplement: Supplementary file 1 [file Data_Sheet_1.PDF]

Supplementary Material

1 Supplementary Data

The following supplementary data will be deposited in Zendo (https://doi.org/10.5281/zenodo.6012529).

- Supplementary_File_1: Movie 1 LAT scan of *Butomus umbellatus*
- Supplementary_File_2: Movie 2 Three-dimensional reconstruction of *Butomus umbellatus* inflorescence
- Supplementary_File_3: Movie 3 Three-dimensional reconstruction of *Butomus umbellatus* vasculature
- Supplementary_File_4: *Butomus umbellatus* vasculature composite tiff file (Can be opened in FIJI)
- Supplementary_File_5: LAT scan of O*rnithogalum umbellatum*
- Supplementary_File_6: *Ornithogalum umbellatum* vasculature tiff file (Can be opened in FIJI)
- Supplementary_File_7: LAT scan of *Allium hollandicum* inflorescence

2 Supplementary Figures

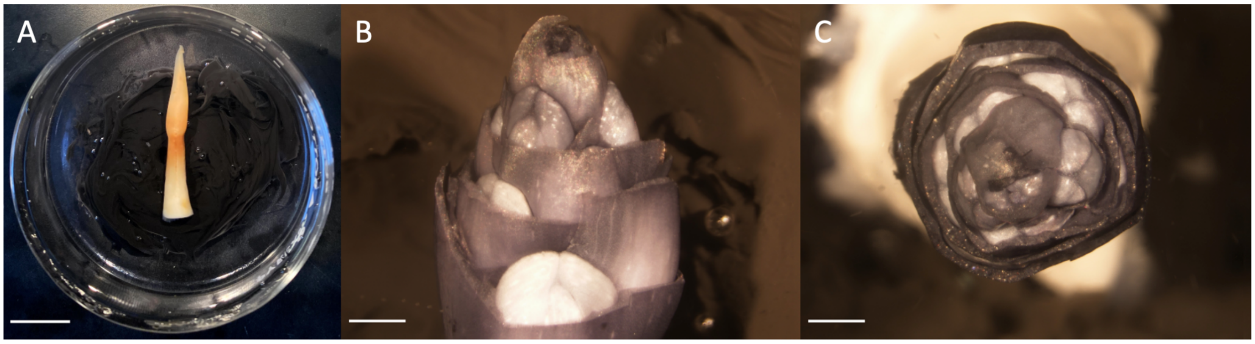

Supplementary Figure 1. Samples examined with laser ablation tomography under

A) Sample *B. umbellatus* reconstructed in Fig. 10. **B-C)** Sample of *O. umbellatus* reconstructed in Fig. 11 imaged, stained with Nigrosin, imaged laterally **(B)** and apically **(C).** Scale bar is 0.5mm in all images

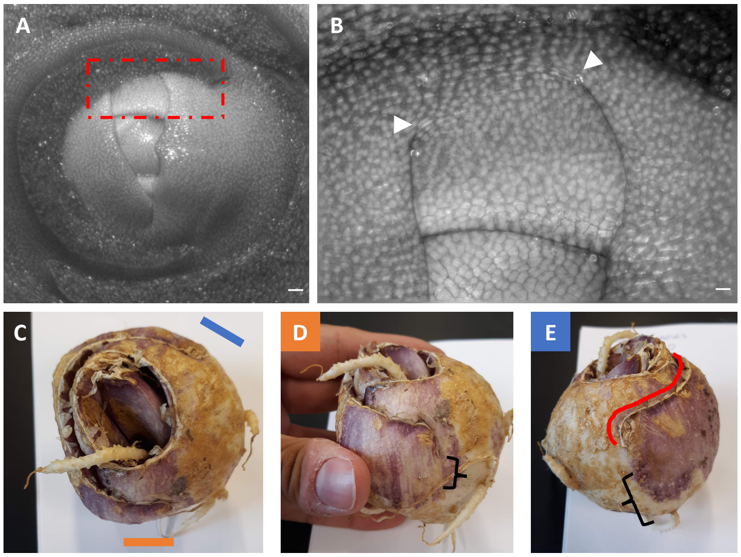

Supplementary Figure 2. Congenital fusion in *Fritillria persica* bulb

A-B) Young *F. persicaia* meristem showing proximal congenital fusion of bulb leaves. This meristem is the same as in Fig. 7A. **(B)** is a close-up of the red dashed box in **(A). C-E**) Mature bulb imaged apically **(C)** and laterally **(D)** and **(E).** Colored lines in **(C)** indicate orientation of the inflorescence in subsequent panels. Black bracket in **(D-E)** indicate leaf fusion. Red line in **(E)** indicate unfused margin of leave.

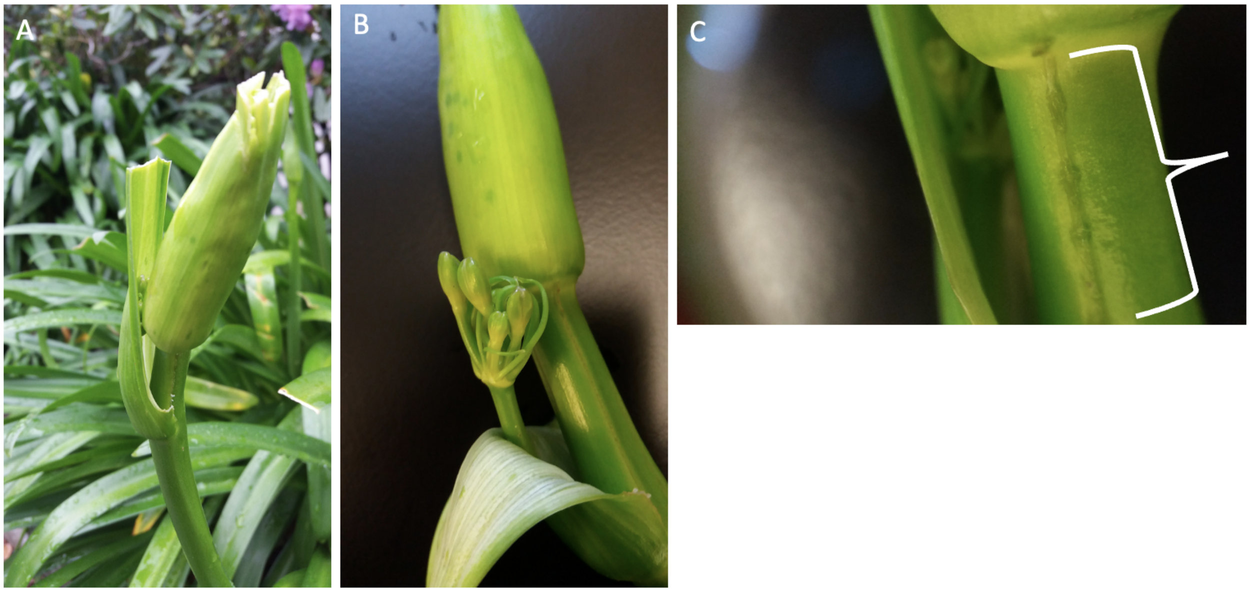

Supplementary Figure 3. ***Agapanthus*** **aberrant inflorescence phenotype**

A) Apparent phenotype located on the plant **B)** Bract exposing rudimentary umbels. **C)** Zoom in of panel B. White bract denotes scarring.
